# Hydrogel-Based Nitric Oxide Delivery Systems for Enhanced Wound Healing

**DOI:** 10.3390/gels11080621

**Published:** 2025-08-08

**Authors:** Tae-Hyun Heo, Hye-Jeong Jang, Gun-Jae Jeong, Jeong-Kee Yoon

**Affiliations:** 1 Department of Systems Biotechnology, Chung-Ang University, Anseong-si 17546, Republic of Korea; 2 Institute of Cell and Tissue Engineering, College of Medicine, The Catholic University of Korea, Seoul 06591, Republic of Korea

**Keywords:** angiogenesis, nitric oxide, hydrogel, reactive oxygen species, wound healing

## Abstract

Oxidative stress in hypoxic conditions impairs the regenerative process in chronic wounds, highlighting the potential of reactive oxygen species (ROS) scavengers to accelerate wound healing. Nitric oxide (NO) in particular plays a pivotal role as an endogenous gasotransmitter and as a signaling molecule involved in regulating hypoxia. In this review, we examine hydrogel-based wound healing strategies for delivering gaseous NO molecules stably to the wound site. As carriers of NO donors, these hydrogels facilitate the controlled and sustained release of NO and offer high biocompatibility and hydrophilicity. First, we first introduce the hypoxic physiology of chronic wounds and elucidate the beneficial and detrimental effects of ROS. In addition, we discuss the role of NO in angiogenesis and the wound healing process. Finally, we review various NO donors and their incorporation into hydrogels for therapeutic applications. Given the extensive use of hydrogels in wound healing, this review will provide valuable avenues for the consideration of new functional hydrogels in regenerative treatments.

## 1. Introduction

Skin wounds are defined as structural disruptions of the skin barrier caused by external factors, leading to the compromise of its essential protective functions [1,2,3]. These wounds are broadly classified as either acute or chronic based on their duration and healing behavior. In recent years, chronic wounds such as pressure ulcers, diabetic ulcers, and venous leg ulcers have become increasingly prevalent. Unlike acute wounds that typically follow a predictable healing process, chronic wounds, or acute wounds exceeding the critical defect size, lose their intrinsic regenerative capacity and fail to progress through normal healing stages. In such cases, therapeutic interventions including skin grafts or engineered biomaterials are necessary to facilitate wound closure with minimal abnormal tissue formation [4,5]. Wound healing is a complex, multi-phase process that involves hemostasis, inflammation, proliferation, and remodeling phases. In the early stages, particularly during inflammation and proliferation phases, elevated levels of reactive oxygen species (ROS) are commonly observed. While ROS are essential for initiating defense and signaling pathways, excessive oxidative stress can exacerbate tissue damage and impair healing, representing a double-edged sword in the regenerative context [6]. Thus, therapeutic strategies that both regulate ROS levels and address the hypoxic microenvironment through angiogenic approaches have drawn significant attention in efforts to enhance skin regeneration.

Various angiogenic factors including growth factors, such as vascular endothelial growth factor (VEGF), fibroblast growth factor (FGF), platelet-derived growth factor (PDGF), and transforming growth factor beta (TGF-β), are currently being investigated [7]. In spite of their high angiogenic efficacy, their poor in vivo stability, limited bioavailability, and concerns over tumorigenicity present significant hurdles to their clinical use, which have prompted the exploration of alternative molecules with safer and more controllable profiles [8,9]. Recently, nitric oxide (NO), a gaseous signaling molecule and member of the gasotransmitter family alongside carbon monoxide (CO) and hydrogen sulfide (H_2_S), has emerged as a compelling candidate. NO not only regulates vascular tone and angiogenesis but also acts as an ROS scavenger, offering multimodal benefit in the oxidative and hypoxic microenvironment of chronic wounds [10]. Due to its small molecular size and uncharged property, NO freely diffuses across cell membranes without requiring transporters [11,12,13]. However, its short biological half-life, lack of targeted delivery, and difficulties in achieving sustained release have posed challenges for therapeutic application [14,15].

**Figure 1:**
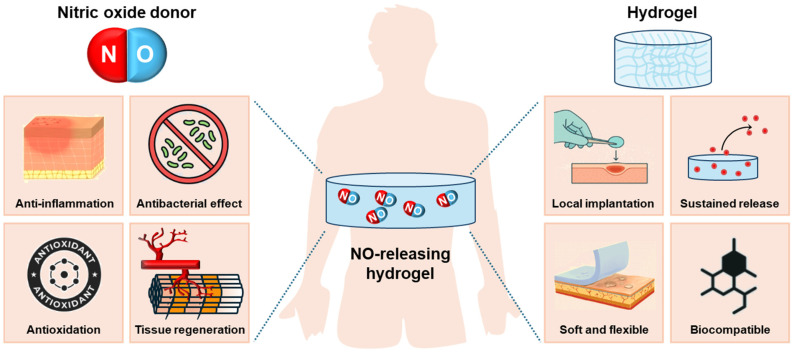
Schematic illustration of the role of nitric oxide (NO)-releasing hydrogel for wound healing. The NO-releasing hydrogel is formed by integrating NO donors and hydrogel matrices to enable localized and sustained NO delivery at the wound site. NO exerts multiple therapeutic effects, including anti-inflammation, antibacterial effect, antioxidation, and tissue regeneration including angiogenesis and ECM remodeling. Hydrogels serve as biocompatible carriers with favorable mechanical properties for local drug delivery and sustained release.

To overcome these hurdles, hydrogel-based NO delivery systems have gained attention as a promising platform (Figure 1). Hydrogels are hydrophilic, biocompatible materials capable of retaining large amounts of water, thereby maintaining a moist wound environment that is widely applied to tissue repair [16,17]. In addition, their tunable softness and elasticity allow them to conform to diverse wound geometries, improving contact and patient comfort to maximize the transepithelial drug delivery efficiency [18,19]. Hydrogel matrices have also been formulated from a variety of natural sources, including food-derived proteins with wound-compatible rheological properties [20]. Beyond serving as drug carriers, functionalized hydrogels actively participate in the therapeutic processes. For example, stimuli-sensitive hydrogels respond to the pH, temperature, or enzymatic activity of the tissue microenvironment, triggering the release of therapeutic agents along with the change in the polymeric structure [21]. Also, hydrogels can be functionalized with cell adhesion peptides (e.g., RGD) or growth factor-mimetic domains to induce cellular recruitment, proliferation, and angiogenesis, further promoting tissue regeneration [22]. Particularly, some hydrogels are engineered to scavenge excess ROS through the incorporation of antioxidant moieties within the polymeric structure [23]. For instance, hydrogels containing NO donors facilitate NO generation, either during their crosslinking or upon application to the wound site, for in situ NO release at the wound lesion. This review provides an overview of the pathological role of ROS in chronic wounds and highlights the therapeutic relevance of NO. It further explores the design principles and mechanisms of hydrogel-based NO delivery systems, offering insight into their translational potential and future prospects in wound healing therapies.

This review summarizes recent advances in nitric oxide-releasing hydrogels, with a particular focus on their classification according to activation mechanisms, namely enzymatic and non-enzymatic systems. In addition to the chemical composition of NO donors, our work highlights how activation mechanisms can be strategically aligned with distinct pathological features of chronic wounds, such as high oxidative stress, hypoxia, and aberrant enzymatic activity. This function-oriented perspective provides a practical approach for tailoring NO-based therapies to specific microenvironments. Furthermore, this classification also extends to broader contexts of inflammatory diseases and tissue regeneration, offering insights into how dynamic microenvironment-responsive hydrogels can be translated toward precision medicine in regenerative applications. The relevant literature was systematically identified through targeted searches in PubMed, Scopus, Web of Science, and Google Scholar, combining keywords, such as “nitric oxide,” “NO donor,” “hydrogel,” “wound healing,” “angiogenesis,” and “oxidative stress”. Peer-reviewed articles from 2010 to 2024 were prioritized, and foundational earlier studies included where relevant.

## 2. Pathology of Chronic Wounds

Chronic wounds arise from a failure of the normal healing cascade, often due to persistent hypoxia, dysregulated inflammation, and microbial colonization. Unlike acute wounds, which naturally heal through hemostasis, inflammation, proliferation, and remodeling in a timely manner, the regenerative cascades of chronic wounds are impaired and typically stalled in the inflammatory phase. Among the various pathological features, oxidative stress induced by the hypoxic microenvironment plays central roles in initiating and sustaining the dysfunctional wound environment [24,25,26]. Hypoxia disrupts tissue homeostasis by increasing ROS production and activating pro-inflammatory signaling pathways. In turn, prolonged infection and microbial colonization further consume oxygen and promote biofilm formation, which exacerbates inflammation, impairs angiogenesis, and hinders extracellular matrix (ECM) remodeling, followed by restricted nutrient delivery, delayed cell proliferation, and inhibited tissue repair [27,28,29].

### 2.1. Hypoxia in Chronic Wounds

The oxygen level is a critical regulator of the wound healing process, influencing cell survival, angiogenesis, collagen deposition, and immunoregulation during the regenerative cascade [30]. In chronic wounds, sustained hypoxia disrupts these cellular functions by impairing oxygen-dependent cellular metabolism, increasing acidosis and altering local immune responses [31,32,33,34].

Hypoxia-inducible factor-1 (HIF-1) is a transcription factor that regulates cell survival under hypoxic conditions. Activation of the HIF-1 signaling cascade increases angiogenic growth factor release, including VEGF, angiopoietin-2, and stromal cell-derived factor-1 (SDF-1), which promote angiogenesis and tissue remodeling [35,36,37]. However, in chronic wounds, prolonged hypoxia affects fibroblasts, keratinocytes, and endothelial cells, leading to inadequate angiogenesis despite HIF-1 activation [38]. The delayed vascularization fails to restore sufficient oxygen levels, resulting in a continued reliance on anaerobic metabolism, ATP depletion, and tissue acidification [39]. These metabolic disruptions and chronic inflammation induce a pathological imbalance and hinders tissue regeneration [39]. Therefore, therapeutic strategies that restore oxygen homeostasis and enhance angiogenesis, such as nitric oxide (NO)-based treatments, are essential to reinitiate healing.

### 2.2. ROS in Chronic Wounds

ROS are key signaling molecules in the wound healing process, but their role is highly dose- and context-dependent. At physiological levels, ROS support host defense and drive regeneration by stimulating keratinocyte migration, fibroblast activation, collagen synthesis, and angiogenesis [40,41]. These functions are particularly important in the early inflammatory and proliferative stages of healing [42,43,44]. During the early stages of wound healing, ROS levels increase to promote tissue repair and act as secondary messengers to up-regulate the secretion of regenerative growth factors [45,46] (Figure 1). For example, TGF-β promotes fibroblast activation and collagen synthesis, and VEGF induces angiogenesis [47,48,49]. In addition, the platelet-derived growth factor (PDGF) recruits and activates fibroblasts and macrophages [50,51]. Epidermal growth factor (EGF) influences angiogenesis and epithelial cell proliferation and migration during wound healing [52]. In addition, ROS initiate the production and the release of pro-inflammatory cytokines such as interleukin-1 (IL-1) and tumor necrosis factor-alpha (TNF-α), both of which promote immune cell-mediated wound healing [53,54,55].

However, deviations from physiological ROS levels are detrimental to wound healing, as insufficient ROS can cause cell cycle arrest and impair signaling required for regeneration, while excessive ROS promote chronic inflammation and oxidative tissue damage [52,56]. Overexpression of ROS in chronic wounds can activate the NF-κB pathway, leading to persistent tumor necrosis factor-alpha (TNF-α) expression, which promotes inflammation and suppresses repair mechanisms [53,55,57,58,59]. Prolonged elevation of TNF-α causes chronic inflammation, disrupts the balance of the repair process [59,60], triggers the NLRP3 inflammasome and increases interleukin-1β (IL-1β) release, contributing to a non-resolving wound microenvironment [61,62]. Importantly, ROS interact with HIF-1 signaling in a dose-dependent manner [63,64]. While moderate ROS levels enhance HIF-1α stability via the PI3K/Akt pathway, excessive ROS accelerate HIF-1α degradation through proteasomal pathways and impair mitochondrial function [65,66]. This dual effect further illustrates the complexity of ROS regulation in wound healing.

Maintaining ROS at a physiological level is, therefore, essential to orchestrate the healing response while preventing oxidative tissue injury. Therefore, ROS-targeted strategies, such as antioxidant delivery, ROS-scavenging materials, or NO-based therapeutics, can help reestablish redox balance in chronic wounds and enable a return to a regenerative trajectory.

## 3. The Role of NO in Wound Healing

NO is a gaseous signaling molecule with diverse biological functions, including vascular regulation, immunoregulation, and tissue regeneration [67]. Synthesized endogenously by nitric oxide synthases (NOS), NO is highly diffusible and plays a pivotal role in modulating wound healing, particularly under the hypoxic and oxidative stress conditions often seen in chronic wounds [68]. This section explores the multifaceted contributions of NO to wound healing, particularly in relation to oxygen homeostasis, redox regulation, and tissue remodeling. Understanding the physiological role of NO may provide the essential fundamentals for the development of therapeutic NO delivery platforms.

### 3.1. NO in Oxygen Homeostasis and Hypoxia Adaptation

Hypoxia is one of the critical factors contributing to chronic wounds, where impaired vascularization leads to insufficient oxygen supply [69]. The oxygen deficiency compromises mitochondrial function and disrupts cellular homeostasis, leading to decreased ATP production. NO plays a critical role in promoting vascular adaptation and cellular survival under these hypoxic conditions.

Hypoxia induces the up-regulation of endothelial NOS (eNOS) expression to enhance NO production from L-arginine [70]. This enzymatic conversion, which requires oxygen, NADPH, and cofactors such as tetrahydrobiopterin (BH_4_), flavin adenine dinucleotide (FAD), and flavin mononucleotide (FMN), yields L-citrulline and NO [71]. The resulting NO activates the soluble guanylyl cyclase (sGC)–cyclic GMP (cGMP) signaling pathway in vascular smooth muscle cells, promoting vasodilation and improved oxygen redistribution to ischemic tissue [72].

NO also facilitates hypoxia adaptation by stabilizing HIF-1, especially by inhibiting prolyl hydroxylase activity, which targets HIF-1α for degradation [73]. Therefore, the prolonged HIF-1 stability enhances the transcription of angiogenic factors such as VEGF, angiopoiten-2, and SDF-1, promoting neovascularization and the recruitment of endothelial progenitor cells to wound sites [74].

At the metabolic level, NO influences the expression of genes associated with energy metabolism, including AMP-activated protein kinase (AMPK), which is a regulator of cellular energy balance [75]. AMPK activation promotes glucose uptake, suppresses energy-consuming anabolic processes, and enhances catabolic pathways to support ATP conservation [76,77,78]. These metabolic shifts support cell survival and maintain cellular function under hypoxic stress.

### 3.2. NO in Redox Balance and Inflammation Regulation

While reactive oxygen species (ROS) and nitric oxide (NO) both act as signaling molecules in the wound environment, their downstream effects diverge significantly. ROS are essential for initial defense responses but become detrimental when overproduced, triggering chronic inflammation and tissue damage. In contrast, NO acts as a scavenger of excess ROS, enhancing antioxidant enzyme activity and modulating key inflammatory signaling pathways. These actions contribute to the restoration of redox and immune balance, thus creating a regenerative microenvironment.

NO reacts with superoxide anions (O_2_) to form peroxynitrite (ONOO^−^), a reactive nitrogen species (RNS) that mitigates oxidative stress and prevents damage to lipids, proteins, and nucleic acids [79]. In addition, NO enhances antioxidant enzymes activity such as superoxide dismutase (SOD) and catalase. SOD catalyzes the conversion of superoxide into hydrogen peroxide, which is subsequently broken down by catalase into water and oxygen, further stabilizing redox balance [80]. Peroxynitrite, while protective in terms of ROS detoxification, also contributes to host defense. In macrophages and neutrophils, NO production via inducible NOS (iNOS) leads to the peroxynitrite generation, which exerts cytotoxic effects against pathogens through oxidative and nitrative damage to microbial membranes, proteins, and nucleic acids [81,82]. In parallel, NO modulates inflammatory signaling by inhibiting the NF-κB pathway. This promotes macrophage polarization toward an anti-inflammatory M2 phenotype and suppresses the production of pro-inflammatory cytokines [83]. These immunomodulatory effects help resolve chronic inflammation and facilitate the transition toward the proliferative phase of wound healing. Consequently, the redox balance via NO further highlights the antibacterial and anti-inflammatory properties by inhibiting the excessive oxidative stress induced in the chronic wound lesion.

### 3.3. NO in Cellular Regeneration and Tissue Remodeling

Once redox balance is restored and inflammation is resolved, the wound healing process advances into the proliferative and remodeling phases. In these stages, NO plays a key role in regulating cellular events, such as endothelial proliferation, keratinocyte migration, fibroblast activation, and ECM remodeling.

During the proliferation phase, NO interacts with VEGF, FGF, and EGF to promote endothelial cell proliferation and angiogenesis [84,85]. NO amplifies VEGF signaling by up-regulating VEGF receptor expression and phosphorylation [86]. In addition, as discussed earlier, NO stabilizes HIF-1α in hypoxic conditions, which increases the production and secretion of VEGF [87]. NO also enhances EGF signaling by promoting epidermal growth factor receptor (EGFR) phosphorylation, which facilitates keratinocyte proliferation and migration through MAPK and PI3K/Akt pathway activation, contributing to re-epithelialization and wound closure [88,89,90,91].

In the remodeling phase, which is the final stage of wound healing, NO supports ECM maturation and scar tissue formation to restore the biomechanical properties of the skin tissue. Fibroblasts, as key effectors of tissue remodeling, increase collagen production under NO stimulation [92,93,94]. In addition, NO induces ECM turnover by up-regulating the activity of MMPs through S-nitrosylation to prevent scar tissue formation [95,96]. The balance between MMPs and tissue inhibitors of metalloproteinases (TIMPs) is also regulated by NO, which contributes to the controlled degradation and synthesis of ECM proteins [97]. NO also promotes myofibroblast differentiation and contractility, thereby contributing to wound contraction and improved tissue integrity [98].

## 4. NO-Releasing Hydrogels for Wound Healing

Due to the short half-life and high diffusivity of nitric oxide (NO), its direct administration to localized wound sites is challenging. Thus, biomaterials have been explored to enable sustained and targeted NO release. Among them, hydrogels have emerged as one of the most promising delivery platforms. Hydrogels are hydrophilic, biocompatible polymers capable of absorbing significant amounts of water while maintaining their structural integrity [99]. This high water content provides a moist wound environment, which is essential for re-epithelialization, cell migration, and nutrient diffusion. In addition, their soft and elastic properties allow for conformal coverage of irregular and dynamic wound surfaces, improving contact and comfort during application. A wide range of natural and synthetic polymers have been used to formulate hydrogels for wound healing applications. Natural polymers, such as hyaluronic acid, gelatin, alginate, and chitosan, are frequently employed due to their inherent biocompatibility, biodegradability, and bioactivity [100]. Synthetic polymers such as polyethylene glycol (PEG), polyvinyl alcohol (PVA), and Pluronic F-127 offer tunable mechanical properties and improved batch-to-batch reproducibility [101]. These materials can be used individually or in combination to optimize hydrogel performance for specific clinical needs.

In the context of wound healing, hydrogels have demonstrated efficacy in not only maintaining a favorable healing microenvironment but also serving as carriers for therapeutic agents. Among these, NO-releasing hydrogels have demonstrated efficacy not only in chronic wounds but also in acute injuries and infected lesions [102]. This therapeutic versatility is attributed to the multifaceted roles of NO, including antibacterial activity, pro-angiogenic stimulation, and immunomodulation [103,104]. These properties are beneficial across all phases of wound repair, regardless of wound chronicity.

To facilitate NO delivery, these hydrogels incorporate NO donors, which are the compounds capable of releasing NO in response to physiological or environmental triggers [105]. Various NO donors are incorporated to serve as stable sources of NO, which can be activated by biochemical or environmental cues. Similar to other hydrogel-mediated drug delivery systems, the release profile of NO or NO donors can be finely tuned by modifying the hydrogel composition, crosslinking density, and porosity [106,107]. This section classifies NO-releasing hydrogels based on donor types and activation mechanisms and highlights representative systems tailored for wound applications.

### 4.1. NO Donors and Their Integration into Hydrogels

Sustained and controlled NO delivery via gaseous administration remains technically challenging due to difficulties in dose regulation and storage stability [108]. To overcome these limitations, various NO donors have been applied to enable consistent in situ NO generation and release [109]. The selection of NO donors arises from their mechanistic reactions, tissue specificity, and concentration dependence [110,111].

L-arginine, the natural substrate for NOS, is enzymatically converted to NO through an oxygen- and cofactor-mediated reaction that generates L-citrulline and NO via a two-step monooxygenase process. S-nitrosothiols (RSNOs), such as S-nitrosoglutathione (GSNO) and S-nitroso-N-acetylpenicillamine (SNAP), release NO through homolytic cleavage of the S-NO bond, triggered by light, heat, or transition metals [107]. These compounds are advantageous for targeted NO delivery due to their chemical stability and ability for controlled release. Nitrites (NO_2_^−^) and nitrates (NO_3_^−^) are precursors of NO in biological systems. While nitrites release NO under acidic or hypoxic conditions, nitrates require bacterial or enzymatic reduction to form nitrites first [112]. These pathways are particularly active in ischemic or infected tissues [113]. Diazeniumdiolates (NONOates) are synthetic NO donors that spontaneously release NO in aqueous solution through proton-driven decomposition, yielding two moles of NO per molecule [114]. Their NO release kinetics can be controlled by chemical modifications depending on their therapeutic purposes and could be engineered for light-triggered release [115]. Nitrosamines, light-sensitive NO donors, have advantages including ease of synthesis and good chemical stability [116]. For example, N,N’-di-sec-butyl-N,N’-dinitroso-1,4-phenylenediam (BNN6) was developed to release NO via 470 nm (blue-ray) irradiation in a controlled release manner with high precision [117]. This photoinduced NO release method offers a non-invasive method for therapeutic applications [118].

Incorporating NO donors into hydrogels offers key advantages for therapeutic applications. The hydrogel network enhances the chemical stability of NO donors and enables localized, sustained release at the wound site, minimizing off-target effects. This controlled delivery is particularly beneficial in wounds requiring prolonged angiogenic and anti-inflammatory support. Moreover, the synergistic combination of hydrogels and NO donors leads to multifactorial benefits. For example, the hydrogel maintains a moist, biocompatible environment, while NO actively promotes vascularization, reduces microbial burden, and supports tissue regeneration. Together, these effects accelerate wound closure and improve overall healing outcomes, highlighting the potential of NO-releasing hydrogels as an advanced wound healing strategy.

### 4.2. NO-Releasing Hydrogels

As discussed in the previous section, NO donors are activated through specific mechanisms that can be broadly categorized into enzymatic and non-enzymatic systems. Enzymatic systems rely on biological stimuli, such as hypoxia or inflammation, to initiate NO release in a manner that mimics endogenous NO production pathways [119]. In contrast, non-enzymatic systems utilize physicochemical triggers, including temperature, light, or changes in environmental pH, to induce NO release from donors, such as GSNO, SNAP, BNN6, and NONOates [102]. This classification framework provides a useful basis for designing hydrogel-based platforms tailored to wound microenvironments, allowing for the regulation of NO release kinetics and its local delivery (Table 1).

#### 4.2.1. Enzymatic NO-Releasing Hydrogels

Enzymatic NO-releasing hydrogels offer localized and biologically responsive NO delivery systems by utilizing enzyme-mediated activation pathways. Due to the specificity of endogenous enzymes, these hydrogels enhance therapeutic selectivity and reduce the need for external triggers, which is advantageous as a wound healing scaffold with variable access or limited user control with minimal off-target effects [137].

**Figure 2:**
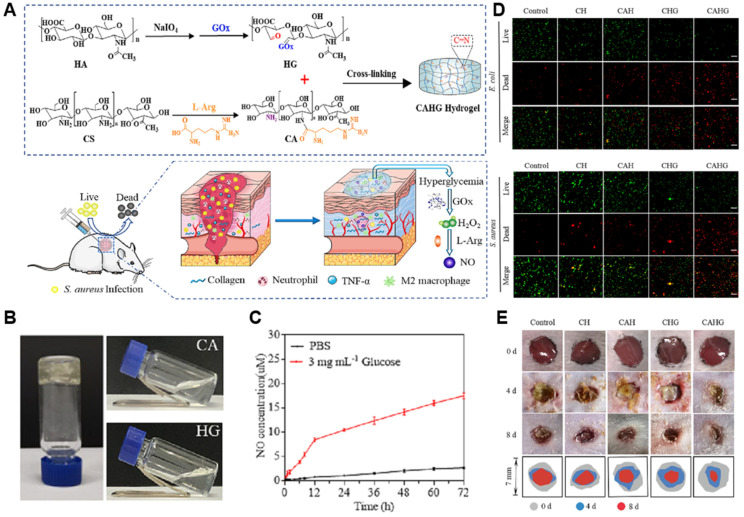
A glucose-responsive NO-releasing hydrogel for treating infected diabetic wounds. (**A**) Schematic illustration of hydrogel synthesis and NO generation. Glucose oxidase (GOx) catalyzes glucose into hydrogen peroxide (H_2_O_2_), which subsequently oxidizes L-arginine to release NO, enabling in situ and glucose-responsive NO generation [120]. (**B**) Photographs of gelling experiments for CA, HG, and CAHG hydrogels [120]. (**C**) Cumulative NO release from the CAHG hydrogel in PBS or 3 mg/mL glucose solution [120]. (**D**) Live/dead staining of Escherichia coli and Staphylococcus aureus after treatment with different hydrogels, highlighting the superior antibacterial performance of CAHG [120]. (**E**) Representative images showing wound healing progression over 8 days in an infected diabetic mouse model treated with each hydrogel formulation [120]. All panels were adapted and reproduced with permission from Xiang et al., Journal of Controlled Release 2023. Copyright © 2025 Elsevier Ltd.

**Figure 3:**
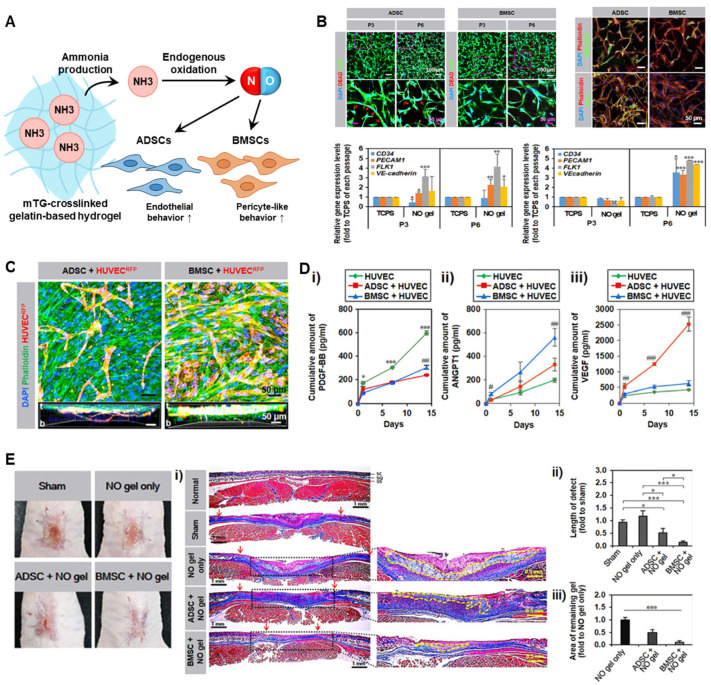
Enzymatic-NO-releasing hydrogels for mesenchymal stem cells (MSCs) engineering. (**A**) Schematic illustration of the mTG-mediated NO generation mechanism within gelatin-based hydrogels which leads EC- and pericyte-like behaviors in ADSCs and BMSCs, respectively. (**B**) Immunofluorescence staining and qPCR analyses of endothelial markers expressed on MSCs cultured within the hydrogel [104]. (**C**) Immunofluorescence staining of MSCs co-cultured with endothelial cells [104]. (**D**) Quantitative ELISA results showing increased secretion of angiogenic factors [104]. (**E**) In vivo wound healing assay of the hydrogel in the presence of MSCs [104] (* *p* < 0.05, ** *p* < 0.01, and *** *p* < 0.001; # *p* < 0.05, ## *p* < 0.01, and ### *p* < 0.001). All panels except A were reproduced with permission from [104]. Distributed under the terms of the Creative Commons Attribution Non-Commercial License 4.0 (CC BY-NC).

For example, a self-healing hydrogel composed of L-arginine-conjugated chitosan with glucose oxidase (GOx)-modified hyaluronic acid was shown to generate both H_2_O_2_ and NO upon glucose exposure (Figure 2) [120]. In this system, GOx catalyzes glucose oxidation to produce H_2_O_2_, which subsequently reacts with the guanidino moiety of L-arginine, leading to non-enzymatic NO generation through oxidative decomposition [120]. The synergistic effects of NO and peroxynitrite released from the hydrogel exhibited robust antibacterial activity against *E. coli* and *S. aureus* without cytotoxicity [120]. Moreover, in a full-thickness skin wound model, the hydrogel significantly accelerated wound healing, achieving approximately 90.5% wound closure by day 8, compared to 65.76–73.20% in various control groups including PBS and single-component formulations [120]. In another study, L-arginine was incorporated into a carboxymethyl cellulose–chitosan matrix to enable enzyme-responsive NO generation [121]. The hydrogel rapidly released NO upon exposure to oxidative conditions, reaching an estimated concentration of ~6 μmol/L within 10 min, as measured by Griess assay. This hydrogel exhibited a robust angiogenic effect via increasing VEGF secretion by ~1.7-fold and promoting tube formation in human umbilical vein endothelial cells (HUVECs) [121]. Furthermore, in a full-thickness rat wound model, CMC-LA/CS hydrogel treatment accelerated healing with an estimated ~95% wound closure by day 14, compared to ~70% in the PBS-treated group, and was accompanied by increased granulation tissue thickness and collagen deposition [121]. A hydrogel composed of gelatin and enzymatically crosslinked by horseradish peroxidase and tyrosinase in the presence of copper ions is another example enzymatic NO-generating approach [122]. This copper nanoparticle hydrogel (GH/Cu) system exhibited a prolonged NO release of up to 180 μM over 7 days, depending on Cu concentration [122]. In vitro assays demonstrated that GH/Cu75 enhanced endothelial cell migration by 40% compared to GH/Cu25 and promoted tube formation with approximately 90% efficacy relative to VEGF control [122]. This system enabled the in situ formation of copper nanoparticles that catalyze the decomposition of endogenous RSNOs in the blood for sustained NO release, promoting wound closure and revascularization in diabetic wound model [122]. Another group has developed a gelatin-based hydrogel crosslinked by microbial transglutaminase (mTG) to investigate the angiogenic effect of NO to human mesenchymal stem cells (hMSCs) derived from bone marrow or adipose tissue (Figure 3) [104]. In this system, mTG catalyzes the formation of isopeptide bonds between glutamine and lysine residues in gelatin, generating ammonia as a by-product, which is subsequently oxidized into NO through an endogenous NO cycle [104]. Colorimetric Griess assays revealed that the hydrogel system released approximately 13 μM of NO over 5 days, indicating a sustained and therapeutically relevant NO delivery profile [104]. NO released from the hydrogel promoted neovascularization supported by its lineage-specific behavior, with bone marrow-derived MSCs exhibiting pericyte-like behavior and adipose-derived MSCs contributing to endothelial cell function and vessel formation [104]. In a murine wound model, BMSC-embedded NO hydrogels accelerated wound closure by day 14, accompanied by enhanced formation of α-SMA+/PECAM1+ vessel formation and increased pericyte marker expression compared to ADSC or gel-only groups [104].

#### 4.2.2. Non-Enzymatic NO-Releasing Hydrogels

Non-enzymatic NO-releasing hydrogels are activated by physicochemical mechanisms such as temperature, light, or spontaneous decomposition. These systems offer precise temporal control under external activation, making them suitable for applications requiring tunable NO delivery profiles without being affected by the endogenous microenvironment.

Thermoresponsive hydrogels are one of the most widely studied stimuli-responsive hydrogels, which are designed to release NO in response to body temperature or locally elevated temperatures from external heat. For example, an NO-releasing injectable hydrogel consisting of Pluronic F-127 and chitosan (CS) loaded with GSNO exhibited sustained NO release and effective antibacterial activity against *Pseudomonas aeruginosa* [134]. This thermosensitive behavior stems from the reverse thermal gelation properties of Pluronic F-127, which forms micellar networks above its critical gelation temperature, remaining in a low-viscosity solution state at room temperature and undergoing rapid gelation at physiological temperature [134]. At 37 °C, the hydrogel released approximately 30 mmol·L^−1^ of NO over 24 h without an initial burst [124]. Antibacterial assays confirmed its efficacy, with both minimum inhibitory concentration (MIC) and minimum bactericidal concentration (MBC) values of 0.5 μg·mL^−1^, corresponding to 1 mmol·L^−1^ of GSNO. Notably, this antibacterial dose did not exhibit cytotoxicity toward mammalian Vero cells [134]. In another approach, NO microbubbles were embedded within a Poloxamer 407 (P407) thermosensitive hydrogel and applied to diabetic wound models, where they enhanced blood perfusion and tripled neovascularization [124]. These microbubbles, composed of a cellulose-based shell encapsulating NO gas, served as a reservoir for localized and sustained NO delivery while facilitating tissue oxygenation and perfusion [124]. This NO-hydrogel system significantly accelerated wound closure in STZ-induced diabetic rats with hindlimb ischemia, reducing the residual wound area to 1.80% on day 11, compared to approximately 15–25% in control groups [124]. Furthermore, the half-life of NO release was prolonged to 1.21 ± 0.13 h, and cumulative NO release reached 80% over 12 h, demonstrating a sustained delivery profile [124]. Quantitatively, CD31^+^ microvessel density increased by over 3-fold, and laser Doppler imaging confirmed significantly elevated blood perfusion at the wound site, supporting the therapeutic angiogenic effect of the formulation [124].

Light-responsive hydrogels provide another form of a non-enzymatic NO-releasing system, enabling spatially and temporally precise control, for example, a dual-crosslinked hydrogel containing BNN6@ZIF-8@PDA nanoparticles, consisting of BNN6 as a NO donor encapsulated within a zeolitic imidazolate framework (ZIF-8), coated with polydopamine (PDA) [125]. In this system, PDA converts NIR light into heat as a photothermal effect, triggering NO release from BNN6 via thermolysis of its N–NO bonds [125]. The hydrogel achieved potent antibacterial efficacy, reducing the viability of *E. coli* and *S. aureus* to below 20% and 5%, respectively, and significantly accelerated wound healing in *S. aureus*-infected diabetic mice, with the wound area reduced to 18.1% by day 7 and complete re-epithelialization observed by day 14 [125]. A similar study suggested a PDA nanosheet-embedded hydrogel crosslinked with BNN6, which exhibited photothermal responsiveness and enhanced collagen deposition, antibacterial efficacy, and tissue regeneration [126]. The PDA nanosheet provided an efficient photothermal conversion through large surface area, enabling uniform heat distribution within the hydrogel upon NIR irradiation [126]. BNN6 functions as a NO donor in response to the localized heat without the need for external chemical triggers, thereby supporting synergistic antibacterial and regenerative effects [126]. This system demonstrated potent antibacterial efficacy, reducing the viability of *E. coli* and *S. aureus* by 98.9% and 99.7%, respectively [126]. In a full-thickness *S. aureus*-infected mouse model, the hydrogel significantly enhanced collagen deposition and tissue regeneration, achieving the smallest wound area (8.2%) by day 7 and near-complete closure by day 14 [126]. Other similar systems incorporating allomelanin nanoparticles-BNN6 or graphene oxide–BNN6 complexes demonstrated comparable antibacterial and regenerative effects under NIR exposure to induce the antibacterial effect, collagen remodeling, and angiogenesis [127,128]. In the allomelanin-BNN6 system, NIR irradiation induced a photothermal response, where the resulting heat activated BNN6 and initiated NO release through cleavage of its N–NO bonds [127]. Similarly, the GO–BNN6 hydrogel used graphene oxide as a photothermal mediator to generate localized heat upon NIR exposure, which in turn promoted NO generation from BNN6 [128].

**Figure 4:**
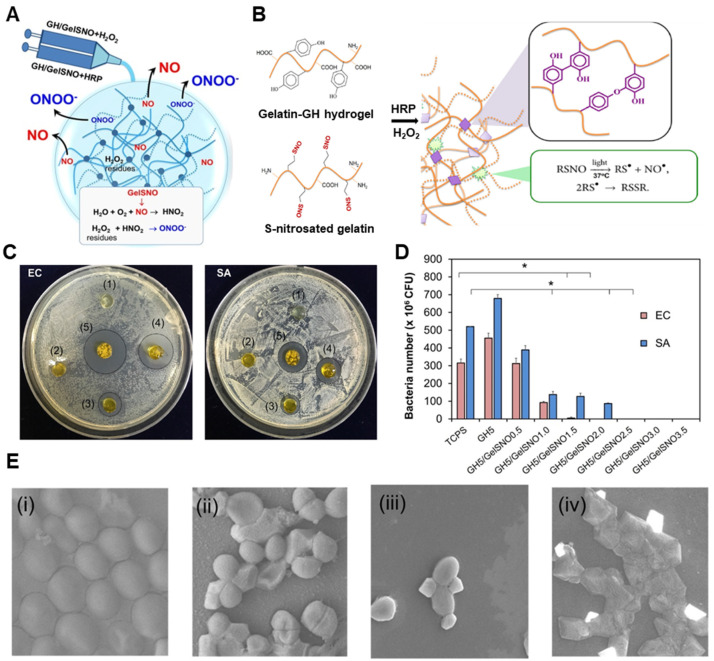
A non-enzymatic NO-releasing hydrogel incorporating S-nitrosothiolated gelatin (GelSNO) as a NO donor for antibacterial application. (**A**) Schematic representation of the NO and reactive nitrogen species (RNS) generation mechanism from GelSNO [129]. (**B**) Chemical structure and crosslinking strategy for the fabrication of the hydrogel [129]. (**C**) Antibacterial effects of the hydrogel against *Escherichia coli* (EC) and *Staphylococcus aureus* (SA) [129]. (**D**) Quantitative analysis of bacterial reduction demonstrating decreases in colony-forming units (CFUs) for both EC and SA depending on the hydrogel formulations (* *p* < 0.05). (**E**) SEM images showing pre- and post-hydrogel treatment on SA (i,ii) and EC (iii,iv) [129]. All panels reproduced with permission from [128], Copyright © 2025 Elsevier Ltd.

Hydrogels based on spontaneously decomposing donors such as RSNOs and NONOates also represent a non-enzymatic approach, offering passive yet sustained NO release without the need for external triggers. For instance, a gelatin-based hydrogel incorporating S-nitrosothiolated gelatin (GelSNO) was developed for prolonged and tunable NO delivery over 14 days, with cumulative release ranging from 0.28 to 2.05 μmol/mL depending on the GelSNO concentration (Figure 4) [129]. The hydrogel exhibited dose-dependent antibacterial effects, where complete inhibition of *E. coli* and *S. aureus* growth was observed at NO levels exceeding 0.39 and 0.58 μmol/mL, respectively [129]. Notably, these antibacterial doses did not compromise cytocompatibility, as over 80% of human dermal fibroblasts remained viable in the presence of NO-releasing formulations [129]. A related study demonstrated that SNAP-loaded chitosan/polyvinyl alcohol (PVA) hydrogels stimulated angiogenesis in chick embryo models and supported BMSC-mediated regenerative responses in diabetic rabbit wounds [130]. Other studies have suggested micelle-embedded hydrogels based on polyethyleneimine (PEI) or poly(acrylic acid) containing NONOates to promote endothelial cell proliferation and modulate vascular smooth muscle activity [131]. In another study, crosslinking of NONOate into an antimicrobial peptide-based hydrogel resulted in a rapid and enhanced bacterial reduction compared to the peptide-only hydrogel [132].

To further expand the therapeutic potential, hydrogels can be integrated with NO donors with additional bioactive agents for synergistic effects. For example, a temperature-sensitive hydrogel composed of brevilin A (BA), camellia oil (CO), and GSNO within a sodium alginate/Pluronic F-127 matrix was formulated in order to enhance the antibacterial and anti-inflammatory effects [133]. This system achieved strong antibacterial effects, reducing multidrug-resistant *Staphylococcus aureus* (MRSA) and multidrug-resistant *Pseudomonas aeruginosa* (MRPA) by up to 99.99%, while maintaining fibroblast viability over 82% at 200 mg/mL. In vivo, the wound area decreased to 5.7% by day 14, compared to 20.7% in untreated mice. The hydrogel also attenuated inflammation, with pro-inflammatory cytokines such as TNF-α and IL-6 reduced by approximately half [133]. Another platform incorporated GSNO and L-arginine in a DNA-inspired hydrogel, leveraging both enzymatic and non-enzymatic NO production pathways to promote vascularized tissue regeneration in diabetic wounds via up-regulation of VEGF and HIF-1α expression [123]. GSNO enabled controlled release, while L-arginine served as a substrate for endogenous NOS to facilitate prolonged NO generation under physiological conditions [123]. Moreover, GSNO-loaded alginate/pectin/PEG hydrogels were developed to enhance cutaneous wound healing through infection control and tissue repair, which are also applied onto vascular stents to prevent neointimal hyperplasia and enhance re-endothelialization, highlighting the versatility of NO-releasing hydrogels beyond dermal applications [135,136].

## 5. Conclusions and Future Prospects

NO-releasing hydrogels combine the multifaceted biological activity of NO with the physical benefits of a hydrated polymer network. The experimental results from in vitro studies and in vivo wound models consistently show therapeutic effects in re-epithelialization, angiogenesis, modulation of inflammation, and reduction in bacterial load. These outcomes highlight NO-releasing hydrogels as an emerging option for managing both acute and chronic skin defects, by overcoming the difficulties in delivering gaseous molecules.

Despite growing interest, several hurdles must be addressed to enable clinical translation. One key challenge lies in maintaining NO release within a therapeutic window, as the release profile is highly sensitive to changes in pH, temperature, enzyme activity, and oxygen tension within the wound microenvironment. A previous study reported insufficient release is ineffective, whereas excess NO may delay type I collagen matrix deposition. This underscores the need for smart biomaterials capable of adjusting NO delivery in response to dynamic biochemical cues, as well as co-delivery systems combining NO donors with growth factors, antimicrobial peptides, exosomes, or cell therapy, to address multiple barriers to healing in a single dressing.

In terms of fabrication, the poor long-term mechanical stability of hydrogels will require further optimization for their retention on highly exudative wounds. In addition, the need for scalable and reproducible manufacturing processes, preservation of NO release kinetics during sterilization and storage, and the establishment of clear regulatory pathways should be considered for clinical translation. Developing standardized evaluation protocols and resolving classification issues between drug and device designations will also be essential to advance NO-releasing hydrogels toward clinical applications.

Continued efforts to address these translational barriers will be critical for unlocking the full therapeutic potential of NO-releasing hydrogels in wound care and regenerative medicine. Future progress in material standardization, controlled delivery, and the mechanistic understanding of NO signaling will be essential to advance these systems toward clinically relevant and disease-specific applications.

## Figures and Tables

**Table 1:** Nitric oxide hydrogels in skin repair.

Type	NO Donors	Polymers	Animal Model	Ref
Enzymatic	L-arg	Chitosan with GOx-modified hyaluronic acid	Diabetic mouse	[120]
		Carboxymethyl cellulose/chitosan hydrogel	Rat	[121]
	RSNO	Gelatin-tyramine hydrogel	Mouse	[122]
	Ammonia	Gelatin hydrogel	Mouse, Rat	[104]
	GSNO + L-Arg	DNA-inspired injectable adhesive hydrogel	Rat	[123]
Non-Enzymatic	NO gas (microbubble)	Poloxamer 407 hydrogel	Diabetic Mouse	[124]
	BNN6	PDA	Mouse	[125]
		PDA nanosheet-embedded hydrogel	Mouse	[126]
		Allomelanin-loaded hydrogel	Mouse	[127]
		GO-BNN6 complex hydrogel	Mouse	[128]
	RSNO	Gelatin-hydroxyphenylpropionic acid	-	[129]
	SNAP	Chitosan/PVA hydrogel	Chick embryo	[130]
	NONOate	Micelle-embedded PEI or PAA hydrogel	Rabbit	[131]
		Antimicrobial peptide-based hydrogel	-	[132]
	GSNO	Temperature-sensitive SA/Pluronic F-127 hydrogel	Mouse	[133]
		Pluronic F-127/Chitosan hydrogel		[134]
		Alginate/Pectin/PEG-based in situ hydrogel-forming powder	Mouse	[135]
		Pluronic F127-based hydrogel	Rat	[136]
	GSNO + L-Arg	DNA-inspired injectable adhesive hydrogel	Rat	[123]
